# Gender differences in the impact of psychological distress on methamphetamine use disorder outcomes and treatment effect

**DOI:** 10.1111/add.70315

**Published:** 2026-01-18

**Authors:** Masoumeh Amin-Esmaeili, Himani Byregowda, Ryoko Susukida, Ramin Mojtabai, Rosa M. Crum

**Affiliations:** 1 Department of Mental Health, Johns Hopkins Bloomberg School of Public Health, Baltimore, MD, USA; 2 Department of Psychiatry and Behavioral Sciences, Tulane University School of Medicine, New Orleans, LA, USA; 3 Department of Epidemiology, Johns Hopkins Bloomberg School of Public Health, Baltimore, MD, USA; 4 Department of Psychiatry and Behavioral Sciences, Johns Hopkins University School of Medicine, Baltimore, MD, USA

**Keywords:** comorbidity, gender, mental health, methamphetamine, pharmacological interventions, psychological distress, stimulant use disorders

## Abstract

**Background and aims:**

Methamphetamine use disorder (MUD) is a major public health concern, often complicated by co-occurring psychological distress (PD). Evidence suggests gender differences in both the prevalence of PD and its impact on treatment outcomes. This study examined impacts of PD on MUD treatment outcomes, focusing on gender differences.

**Design:**

Secondary analysis of pooled data from five randomized controlled trials of pharmacotherapy for MUD available on the NIDA DataShare site (accessed 19 October 2024). Individual participant data meta-analysis methods were used, adjusting for sociodemographic factors and accounting for heterogeneity across trials. Regression analyses were conducted for total sample and stratified by gender.

**Setting:**

Treatment facilities in the US.

**Participants:**

Adults seeking MUD treatment (*n* = 866).

**Measurements:**

PD was assessed using the Addiction Severity Index psychiatric domain (≥24.6 cutoff). Outcomes included reductions in methamphetamine use and positive urine tests for methamphetamine and other drugs.

**Findings:**

PD was found in 39.9% of participants. PD was more common among women than men [odds ratio (OR) = 1.56, 95% confidence interval (CI) = 1.15–2.12], and among individuals who were younger (ages 35–45 vs. <35: OR = 0.72, 95% CI = 0.61–0.85; >45 vs. < 35: OR = 0.62, 95% CI = 0.44–0.88), had lower education (OR = 1.38, 95% CI = 1.16–1.65), chronic medical conditions (OR = 1.60, 95% CI = 1.16–2.20), history of injection drug use (OR = 1.47, 95% CI = 1.13–1.91) and prior treatment for alcohol use disorder (OR = 2.52, 95% CI = 1.64–3.84). PD was associated with lower odds of reduced use [adjusted OR (aOR) = 0.74, 95% CI = 0.66–0.82] and higher odds of positive methamphetamine urine tests (aOR = 1.27, 95% CI = 1.08–1.49). Stratified analyses revealed a stronger association among women between PD and lower odds of reduced use (aOR = 0.41, 95% CI = 0.23–0.75) and higher odds of positive urine tests for methamphetamine (aOR = 2.18, 95% CI = 1.25–3.81) and other drugs (aOR = 3.16, 95% CI = 1.53–6.47), whereas men showed no statistically significant impact of PD on treatment outcomes. A statistically significant interaction between treatment, gender and PD (*P* < 0.001) indicated that women without PD benefited more from treatment than those with PD, a pattern not mirrored in men.

**Conclusion:**

Psychological distress appears to negatively impact outcomes for MUD and the effects of MUD treatment, particularly among women. Integrated psychological interventions, tailored by gender, may enhance treatment efficacy for individuals with co-occurring MUD and psychological distress.

## INTRODUCTION

Methamphetamine use disorder (MUD) is a significant global public health challenge. In 2023, in the United States (US), approximately 0.6% of individuals 12 years old or older reported having MUD in the past year, translating to approximately 1.8 million people [1]. Methamphetamine use is associated with a wide array of adverse outcomes, including cognitive impairment [2], cardiovascular complications [3] and an elevated risk of infectious diseases such as HIV and hepatitis C [4–6]. Despite the substantial burden posed by MUD, effective treatment options remain limited, with no US Food and Drug Administration-approved medications available.

### Psychological distress and MUD treatment complexity

A critical factor complicating the treatment landscape for MUD is the high co-occurrence of comorbid conditions, including psychological distress (PD) [7–11]. PD often manifests by anxiety, depression, psychotic symptoms or violence and is highly prevalent among individuals with MUD [8, 12–15]. Research has highlighted mechanisms linking PD with substance use disorders, demonstrating how distress may exacerbate disorder progression and negatively impact treatment outcomes. Elevated emotional stress has been shown to increase drug cravings in individuals with substance use disorders [16–18]. Depressive symptoms are linked to reinforcing effects of psychostimulant use, heightening susceptibility to substance self-administration [19]. Withdrawal and its associated symptoms can heighten stress with drug use serving as a coping mechanism to alleviate anxiety, ultimately leading to poorer outcomes [20]. Treatment strategies targeting psychiatric symptoms may alleviate PD and enhance substance use disorder treatment outcomes [21–23]. However, many individuals experiencing PD exhibit subthreshold symptoms that do not meet diagnostic criteria. Consequently, they are less likely to perceive a need for treatment or engage in psychiatric care. A deeper understanding of this relationship could inform the development of integrated treatment strategies that simultaneously address substance use and PD, ultimately improving treatment engagement and long-term recovery outcomes.

### Gender differences in MUD and PD among people with MUD

Gender differences in factors associated with MUD, as well as the prevalence and impact of PD among individuals with MUD, have received growing attention [24–30]. Prior studies using the Addiction Severity Index (ASI) or similar measures report higher PD among women than men [30–32]. Studies indicate that women often start using methamphetamine at an earlier age and develop dependence more quickly than men [24]. This ‘telescoping effect’—also observed with alcohol, cocaine and other substances—may stem from biological differences between male and female brains that increase susceptibility to addictive patterns of drug use, as well as the influence of sex hormones [25–28]. Psychological factors, such as higher rates of trauma exposure and caregiving stress, may further accelerate disorder progression and complicate recovery [11, 24]. Studies also indicate that women with MUD are more likely than men to experience heightened anxiety, depressive symptoms and social stigma [24, 29, 30]. Additionally, women often face unique barriers to accessing and adhering to treatment, such as caregiving responsibilities and a lack of gender-sensitive treatment programs [33, 34]. Such challenges can lead to distinct patterns of substance use and associated dysfunction, further complicating treatment outcomes. Despite growing recognition of these issues, research examining the interplay between PD and MUD treatment outcomes, including gender differences, remains limited. Understanding these dynamics is crucial for optimizing interventions.

This study leveraged pooled data from randomized controlled trials (RCTs) for the treatment of MUD to examine the impact of PD on treatment outcomes and investigated gender differences in these effects. By exploring how PD interacts with MUD treatment, we aimed to provide insights that can guide the development of tailored, gender-sensitive treatment programs.

## METHODS

### Sample

This study is a secondary analysis of pooled datasets from five harmonized RCTs investigating pharmacological interventions for the treatment of MUD (total sample = 866) [35–38]. The harmonization procedures have been previously described [39]. Briefly, harmonization involved aligning comparable variables across trials, standardizing coding schemes, reviewing documentation to ensure conceptual equivalence and conducting range and consistency checks for variables to verify data quality. Raw data and associated documents are publicly available on the National Institute on Drug Abuse (NIDA) Data Share website (https://datashare.nida.nih.gov). From an initial pool of 13 studies on pharmacotherapies for MUD available on this site (accessed on 19 October 2024), five RCTs were selected. RCTs in phase 1, feasibility, drug interaction studies and those lacking key measures were excluded (Figure S1). The included trials shared a core set of measures and used similar study designs, eligibility criteria and quality assurance procedures.

Each RCT evaluated the effects of one pharmacological agent, including topiramate [35], modafinil [36], bupropion [37, 40] and ondansetron [38], compared to placebo. All participants received cognitive behavioral therapy to enhance treatment adherence.

### Measurement

PD was measured using the psychiatric domain of ASI, version 5 (ASI-5), hereafter called ASI-Psych. The ASI-Psych composite score was computed based on 11 items. The first seven yes/no items asked participants about a number of psychological symptoms (e.g. serious depression, serious anxiety/tension and serious thoughts of suicide) over a significant period in the past 30 days, excluding those directly caused by drug use. An additional item inquired about the use of prescribed medications for psychological or emotional problems. The remaining three items assessed the subjective rating of the magnitude and importance of psychological problems, which included (1) the number of days in which the participants experienced psychological or emotional problems in the past 30 days (0–30); (2) the extent to which these problems were troublesome; and (3) the importance of treatment for these problems. The last two items were scored ordinally from 0 (‘not at all’) to 4 (‘extremely’). The composite score was calculated using the Composite Scores Manual [41], multiplied by 100 (range = 0–100; higher scores indicate greater severity). A binary measure for significant PD in the past 30 days (any vs. none) was derived using a previously validated cut-off score of 24.6 on ASI-Psych [42]. The ASI has been extensively used and validated in diverse populations, including our prior work, and is a reliable instrument for assessing the severity of psychosocial problems in substance use research [7, 21, 42–49].

Multiple treatment outcomes were harmonized across all included RCTs. The primary outcome was any reduction in the frequency of methamphetamine use. First, we categorized frequency of use based on the drug domain of ASI measured at baseline and again at the end of trial into three levels: no use (0 days), low-frequency use (1–4 days) and high-frequency use (5+ days). Next, following our prior studies [50, 51] and research by Roos *et al.* [46], a binary outcome was created to indicate any reduction in use, defined as a transition from high- to low-frequency use or from any use to abstinence. Participants with stable or increased levels of use were categorized as having no reduction in use.

Other outcomes included having a positive methamphetamine urine test as well as positive tests for other drugs (i.e. cocaine, opioids, benzodiazepines, cannabis and barbiturates) at the trial end. Binary outcomes based on urine tests were created, and treatment success was defined as two negative urine tests at two consecutive assessment points immediately before the primary end point of each trial. Treatment failure was defined as any positive urine test, regardless of the results of the other test at the two assessment points immediately before the primary end point of each trial (e.g. positive + negative or positive + missing). If one test was negative and the other was missing, the outcome was considered as missing, because neither success nor failure could be determined.

Other variables included in the models were: participants’ socio-demographic characteristics (gender, race/ethnicity, employment status, education and marital status), clinical characteristics (history of chronic medical illness, injecting drug use, prior treatment for drug or alcohol use disorders and polysubstance use) at baseline and length of trial, harmonized across the studies.

### Statistical analysis

Analyses were conducted in four steps. First, we described participants’ socio-demographic and clinical characteristics by PD status at baseline using univariable logistic regression analyses. Given evidence of gender differences in subsequent models, we also described baseline characteristics separately for women and men. Second, we compared the PD symptoms from ASI-Psych between women and men, using Fisher’s exact tests for rare events, χ^2^ tests for all other variables and examined treatment retention (completion of active trial treatment phase) by PD status and gender. Third, following established guidelines for one-stage individual participant data meta-analyses [52–54], we evaluated the association between PD (primary binary measure; sensitivity analyses with continuous ASI-Psych score) and three treatment outcomes: reduction in methamphetamine use frequency, methamphetamine-positive urine toxicology at the end of treatment and other drug-positive urine toxicology. Two regression models were used for each outcome. Model A adjusted for sociodemographic and clinical covariates and accounted for within-trial correlation using cluster-robust standard errors at trial level. Model B further addressed missing outcome data using inverse probability weighting (IPW). All analyses were repeated after stratifying by gender because of significant PD × gender interactions. Finally, we tested whether gender and PD jointly moderated treatment efficacy by evaluating treatment × gender × PD interactions and conducted gender-stratified analyses to further characterize differential treatment effects. Additional methodological details, including full model specifications and sensitivity analyses, are provided in the Appendix S1. All analyses were conducted in Stata 18.5 with a pre-defined significance level of *P* < 0.05. This analysis was not pre-registered on a publicly available platform, and therefore, the results should be interpreted as exploratory.

## RESULTS

### Socio-demographic and clinical correlates of PD

Of the total sample (*n* = 866), 11 participants had incomplete ASI-Psych items, making it impossible to compute their ASI-Psych composite scores. Among the remaining participants (*n* = 855), 39.9% experienced serious PD, with higher rates observed in women compared to men (46.7% vs. 35.9%; OR = 1.56, 95% CI = 1.15–2.12, *P* = 0.005). Consistent with this finding, women had significantly higher mean ASI-Psych composite scores than men (24.7 vs. 18.8, *P* = 0.022).

PD was also more prevalent among participants younger than 35 years old, those with less than a high school education (diploma or General Education Development), chronic medical conditions and a history of injecting drug use or prior treatment for alcohol use disorders (Table 1). Gender-stratified analysis revealed distinct profiles of factors associated with PD. Among women, those reporting PD were more likely to be non-Hispanic White compared to other racial/ethnic backgrounds (OR = 1.95, *P* < 0.001) and to have a history of chronic medical illness (OR = 1.94, *P* < 0.001). Among men, PD was significantly associated with younger age (<35 years), high school education or lower (vs. higher education) and a prior history of treatment for alcohol use disorder (Table 1). These findings remained consistent when analyzed using mean ASI-Psych composite scores (Table S1).

Overall, 61.7% completed active treatment phase of the RCTs. Participants with PD had a borderline significantly higher rate of treatment retention than those without PD (63.9% vs. 60.1%, *P* = 0.050). In the adjusted model, after controlling for demographic and clinical covariates and clustering by RCT, participants with PD had significantly higher odds of treatment retention compared with those without PD (adjusted OR = 1.30, 95% CI = 1.05–1.61). There were no significant differences in overall treatment retention between women and men (61.0% vs. 62.0%, *P* = 0.762), nor in the association between PD and treatment retention within either gender. Among women, 64.4% with PD vs. 60.7% without PD completed treatment (*P* = 0.240); among men, the rates were 63.3% and 58.9% (*P* = 0.073), respectively.

### Gender differences in symptoms of PD

**FIGURE 1:**
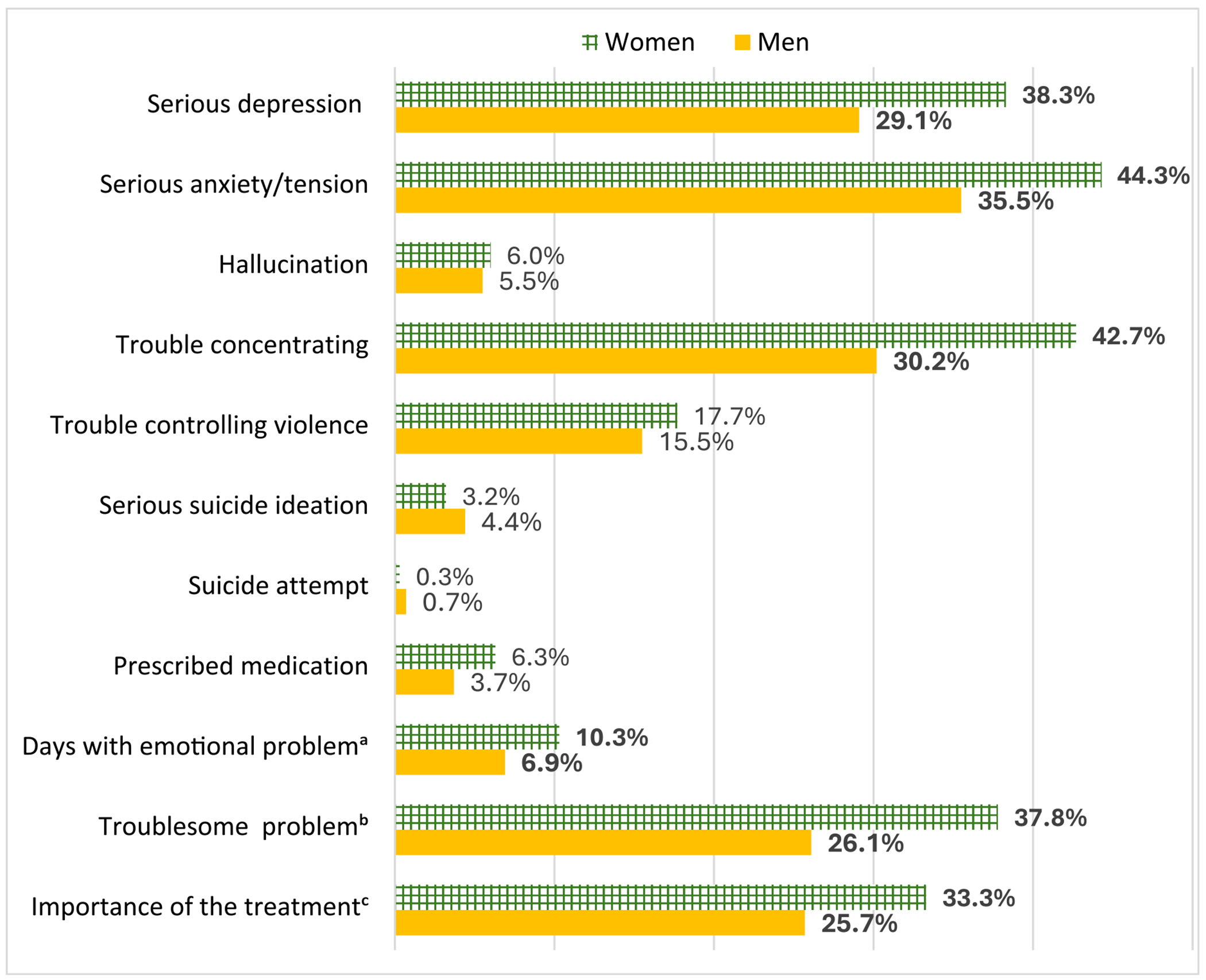
Distribution of symptoms/items of psychological distress (Addiction Severity Index version 5) in the past 30 days by gender. Gender differences in items with bold value labels were statistically significant (*P* < 0.05). ^a^Mean days of experiencing psychological or emotional problems in the past 30 days. ^b^Rated considerably or extremely troublesome emotional problems. ^c^Rated considerably or extremely important treatment is for these emotional problems.

Women reported higher rates of serious depression (38.3% vs. 29.1%, *P* = 0.005), serious anxiety or tension (44.3% vs. 35.5%, *P* = 0.010) and trouble concentrating or understanding (42.7% vs. 30.2%, *P* < 0.001) compared to men (Figure 1). Women also experienced more days with psychological problems in the past 30 days (10.3 days vs. 6.9 days, *P* < 0.001). Furthermore, a higher proportion of women reported that their psychological problems were considerably or extremely troublesome (37.8% vs. 26.1%) and believed that treatment for these problems was considerably or extremely important (33.3% vs. 25.7%, *P* = 0.017).

There were no statistically significant gender differences in the rates of being prescribed psychiatric medication (6.3% in women vs. 3.7% in men, *P* = 0.073) or trouble controlling violent behaviors (17.7% vs. 15.5%, *P* = 0.404). Although men reported slightly higher rates of serious suicidal ideation (4.4% vs. 3.2%, *P* = 0.374) and suicide attempts (0.7% vs. 0.3%, *P* = 0.439) compared to women, these differences were not statistically significant.

### Impact of PD on outcome of treatment for MUD

Analyses of data in both model A and model B (see Table 2) showed significant associations between PD and the outcomes. According to model B, individuals with PD were less likely to achieve reduced methamphetamine use from baseline to the end of trial (OR = 0.74, *P* < 0.001) and more likely to have a positive urine test for methamphetamine at the end of trial (OR = 1.27, *P* = 0.004). A statistically significant interaction between ASI-Psych and gender was observed for achieving any reduced use (*P* = 0.024) and for having a positive test for other drugs (*P* < 0.001). Therefore, stratified analyses by gender were performed to evaluate the impact of PD on treatment outcomes for MUD, separately in men and women. Women with PD were more likely to experience unfavorable outcomes, such as a lower likelihood of reduced use (OR = 0.41, *P* = 0.003), and a higher likelihood of a positive urine test for methamphetamine (OR = 2.18, *P* = 0.006) and other drugs (OR = 3.16, *P* < 0.001) compared to women without PD (Table 2). Men with PD did not significantly differ from those without PD across methamphetamine outcomes and were less likely to have a positive urine test for other drugs (OR = 0.60, *P* < 0.001) (Table 2). The sensitivity analysis results using ASI-Psych composite score as a continuous measure were consistent with our main findings using a binary exposure, although some associations, such as the significant relationship between PD and reduced methamphetamine use among women, were no longer statistically significant (Table S2).

### Gender difference in the impact of PD on MUD treatment effect

No significant interaction between PD and the pooled effect of any pharmacological intervention versus placebo on the study outcomes (two-way interactions). However, the three-way interaction between pooled treatment, PD and gender was significant for all of the outcomes (*P* < 0.001) (Table 3), therefore, results are presented stratified by gender and PD status.

**FIGURE 2:**
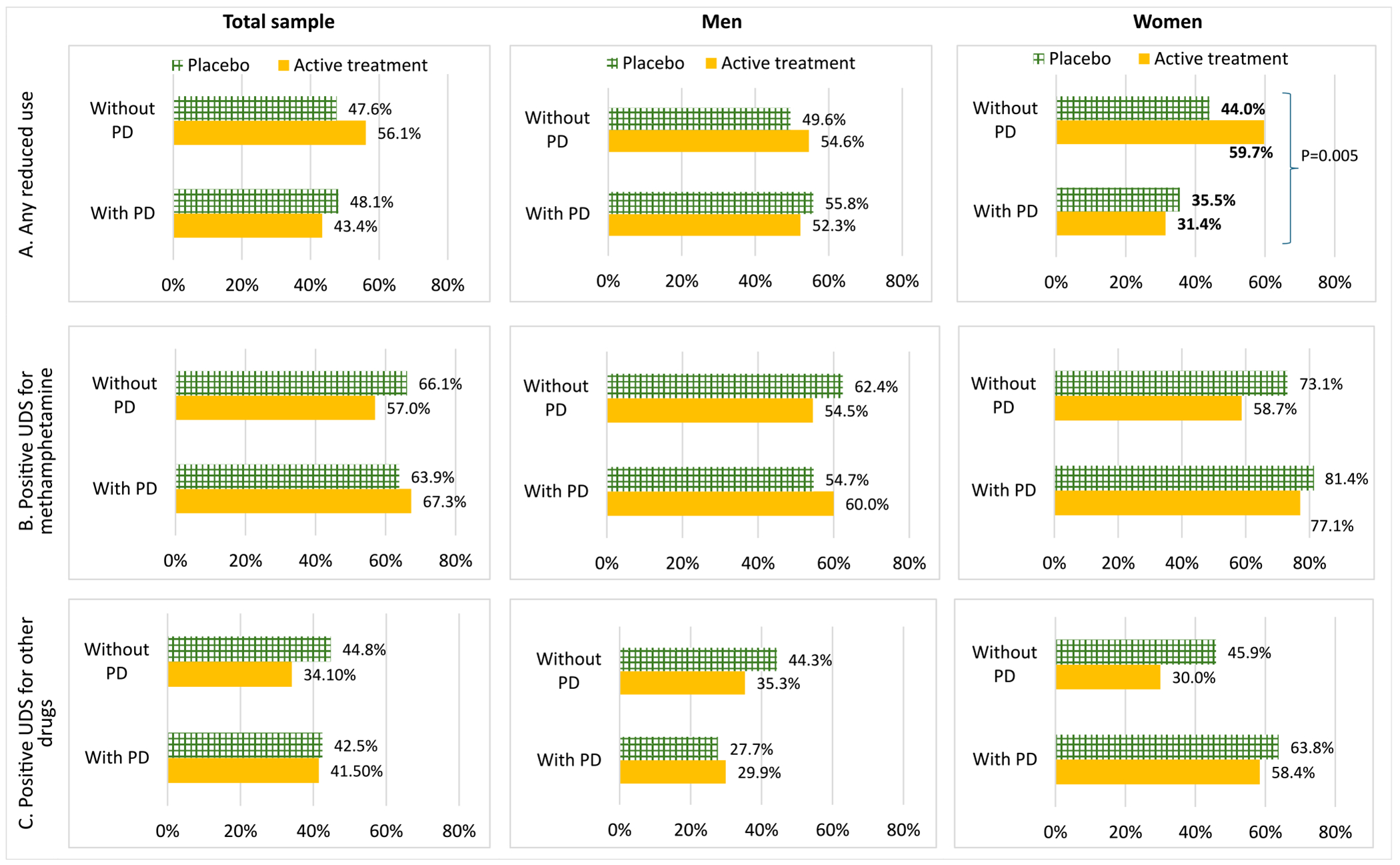
Treatment effect on outcomes of methamphetamine use disorders in those with and without psychological distress, stratified by gender. PD, psychological distress; UDS, urine drug screen. Bars with bold data labels indicate a significant two-way interaction (treatment × PD) in the stratified analysis (*P* = 0.005).

Stratified analyses in subgroups of men and women showed that both women and men without PD benefited more from treatment than from placebo, with women without PD experiencing a particularly marked benefit. Specifically, baseline PD significantly interacted with treatment effects on achieving reduced methamphetamine use among women (*P* = 0.005). Women without PD demonstrated a higher rate of reduced methamphetamine use in treatment arm compared to placebo (59.7% vs. 44.0%), while women with PD exhibited a lower rate of reduced use in treatment arm than in placebo group (31.4% vs. 35.5%) (Figure 2).

We also conducted a sensitivity analysis to control the type of medication in each intervention, which did not significantly alter the findings. In a three-way interaction (gender, PD and treatment), results showed a similar significant interaction for all of the outcomes: any reduced use (χ^2^ = 30.97, *P* < 0.001), positive urine test for methamphetamine (χ^2^ = 21.26, *P* < 0.001) and a positive urine test for other drugs (χ^2^ = 151.38, *P* < 0.001).

## DISCUSSION

The study findings highlight the significant impact of PD on outcomes and treatment effects in individuals with MUD, particularly among women. A key finding is that PD, as assessed by ASI-Psych, was common in the sample and correlated with poorer outcomes overall. The study also revealed significant gender differences in how PD influenced treatment outcomes. Women with PD experienced notably poorer treatment outcomes compared to those without distress.

The finding of high prevalence of PD among individuals seeking MUD treatment is consistent with previous research indicating that PD is prevalent in individuals with substance use disorders [15, 55–57]. A notable gender difference was observed in the prevalence of PD and specific symptoms, consistent with prior studies using ASI and similar measures [30–32, 58, 59]. Women reported higher rates of anxiety, depression and difficulties with concentration compared to men. They also exhibited greater awareness of their PD and a stronger perceived need for treatment, possibly suggesting a higher level of mental health literacy and a more positive attitude toward help seeking for mental health problems than men [60, 61].

These findings underscore the importance of integrating gender-specific considerations into interventions for MUD. Although women may be more attuned to their PD and more likely to seek help, this awareness does not necessarily translate into access to or engagement with appropriate care. Structural and systemic barriers, including financial constraints, stigma, lack of childcare and gender-related disparities in healthcare access, may prevent women from receiving mental health care they need [62, 63]. Conversely, men with PD may be less likely to acknowledge or seek treatment because of cultural norms that discourage emotional vulnerability and helpseeking behavior [61, 63].

Existing literature regarding the impact of comorbid psychopathology on substance use disorders is mixed. However, this literature almost exclusively focused on general substances and certain psychiatric disorders, rather than specifically on the MUD and PD [20, 45, 64–67]. PD, including depressive and anxiety symptoms, can exacerbate drug craving, impair cognitive function and hinder the individual’s ability to engage in treatment, making it harder to achieve positive outcomes. The potential mechanisms through which PD can affect treatment outcomes include heightened emotional reactivity, difficulty concentrating and impaired decision-making, which can impede the process of behavioral change and adherence to treatment protocols [17, 26].

A notable gender difference was also observed in the outcomes of MUD among those with and without PD. Women with PD experienced notably worse outcomes compared to women without distress. They were less likely to achieve reduced methamphetamine use and were more likely to test positive for methamphetamine and other drugs at the end of trial. Sensitivity analyses using the continuous ASI-Psych score yielded largely consistent results, although some associations, such as the relationship between PD and reduced methamphetamine use among women, were attenuated. This likely reflects the nonlinear nature of the PD-outcome relationship rather than instability of the ASI construct or selective reporting, and therefore, should be interpreted with caution. In contrast, men with PD did not experience significantly different outcomes compared to men without distress, with the exception of reduced likelihood of testing positive for other drugs. This may suggest that psychopathology in men is less likely to impact their drug use patterns than in women [33, 45]. These findings imply that women with PD could benefit more than men from interventions that specifically address both their substance use and psychological well-being. Addressing PD in individuals with MUD, particularly women with MUD, may be crucial for improving outcomes.

The findings of this study also underscore the impact of PD on the efficacy of pharmacological interventions, specifically among women. Women without PD benefited significantly from pharmacotherapy, exhibiting a notably higher likelihood of reduced methamphetamine use. In contrast, women with PD appeared to derive less benefit from these treatments. This disparity in treatment response cannot be solely attributed to differences in retention rates. Although PD was associated with slightly higher treatment retention in the overall sample [68], our analysis, consistent with previous research on clinical trials within the National Drug Abuse Treatment Clinical Trials Network [69], found no significant gender differences in treatment retention, nor substantial differences in retention between individuals with and without PD.

Another possible explanation for the impact of PD on efficacy of treatments could be that some medications in the pooled RCTs target both MUD and PD. Insufficient sample sizes, particularly for medications with antidepressant or mood-stabilizing properties, limited our ability to perform medication-specific analyses. However, adjustment for the type of medication did not change the significant three-way interaction. These findings suggest that women with PD experience poorer outcomes with pharmacological treatments for MUD compared to those without PD. Tailored interventions that integrate substance use treatment with mental health support may be necessary to improve outcomes, particularly for women experiencing PD. Moreover, routine screening for PD using validated tools could be implemented in MUD treatment settings. Early identification of distress can inform tailored interventions, potentially mitigating the adverse impact of PD on treatment outcomes.

Given the high prevalence of PD among individuals with MUD and our finding of no relationship between such distress and drop-out, researchers should reconsider the common practice of excluding individuals with PD or psychiatric disorders from clinical trials. Broader inclusion criteria can improve the generalizability of such research.

This study has limitations that should be considered when interpreting the findings. Although the study used the largest sample of individuals with MUD analyzed to date, the limited sample size in some subgroups prohibited further stratified analyses, particularly within medication subgroups. We only examined the association of baseline PD with the outcomes. Future analyses could examine trajectories of PD in the course of treatment and their longitudinal impact on MUD outcomes. Although missing outcome data were addressed using IPW to reduce potential bias because of random missingness, bias from non-random missingness cannot be ruled out. Last, although the included studies were multi-site RCTs recruiting participants from 11 states across the United States, the generalizability of the findings to other US regions or other countries should be considered when interpreting the results.

In conclusion, notwithstanding these limitations, we found that PD significantly impacts outcomes for MUD and the effect of MUD treatment, particularly among women. These findings underscore the critical need for integrated, gender-sensitive interventions that address both substance use and co-occurring PD. By tailoring treatment strategies to the unique needs of individuals with MUD, particularly women, it may be possible to improve recovery outcomes and reduce the burden of this pervasive disorder.

## Supplementary Material

Appendix S1. Supplementary Information on statitiscal analysis

Figure S1. Flowchart of selected RCTs of pharmacotherapies for methamphetamine use disorders (MUD)

Supplementary Tables S1 and S2

Additional supporting information can be found online in the Supporting Information section at the end of this article.

## Figures and Tables

**TABLE 1:** Socio-demographic, clinical and drug use correlates of psychological distress (ASI-Psych ≥24.6) at baseline by gender.

	Total sample				Men				Women			
	*N*	*n* (row%) with PD	OR (95% CI)^a^	*P* value	*N*	*n* (row%) with PD	OR (95% CI)^a^	*P* value	*N*	*n* (row%) with PD	OR (95% CI)^a^	*P* value
Age, years												
<35	316	144 (45.6)	1		197	87 (44.2)	1		119	57 (47.9)	1	
35–45	361	136 (37.7)	0.72 (0.61–0.85)	**<0.001**	223	72 (32.3)	0.60 (0.48–0.77)	**<0.001**	138	64 (46.4)	0.94 (0.72–1.23)	0.653
>45	178	61 (34.2)	0.62 (0.44–0.88)	**0.007**	120	35 (29.2)	0.52 (0.37–0.74)	**<0.001**	58	26 (44.8)	0.88 (0.51–1.54)	0.661
Race/ethnicity												
Other/multiple	252	91 (36.1)	1		179	66 (36.9)	1		73	25 (34.3)	1	
Non-Hispanic White	603	250 (41.5)	1.25 (0.93–1.69)	0.138	361	128 (35.5)	0.94 (0.64–1.39)	0.758	242	122 (50.4)	1.95 (1.37–2.79)	**<0.001**
Unemployed												
No	700	274(39.1)	1		470	169 (36.0)	1		230	105 (45.7)	1	
Yes	154	67 (43.5)	1.20 (0.93–1.54)	0.162	70	25 (35.7)	0.99 (0.61–1.61)	0.966	84	42 (50.0)	1.19 (0.93–1.53)	0.169
Marital status												
Legally married/cohabiting	207	78 (37.7)	1		136	47 (34.6)	1		71	31 (43.7)	1	
Divorced/widowed/separated	287	128 (44.6)	1.33 (0.82–2.17)	0.249	162	66 (40.7)	1.30 (0.76–2.22)	0.331	125	62 (49.6)	1.27 (0.77–2.08)	0.343
Single/never married	361	135 (37.4)	0.99 (0.74–1.32)	0.934	242	81 (33.5)	0.95 (0.89–1.02)	0.147	119	54 (45.4)	1.07 (0.50–2.28)	0.857
Education												
Higher education	379	135 (35.6)	1		235	69 (29.4)	1		144	66 (45.8)	1	
High school/GED^b^ or lower	476	206 (43.3)	1.38 (1.16–1.65)	**<0.001**	305	125 (41.0)	1.67 (1.17–2.39)	**0.005**	171	81 (47.4)	1.06 (0.61–1.84)	0.826
Chronic medical illness												
No	685	258 (37.7)	1		438	151 (34.5)	1		247	107 (43.3)	1	
Yes	169	83 (49.1)	1.60 (1.16–2.20)	**0.004**	102	43 (42.2)	1.39 (0.95–2.03)	0.094	67	40 (59.7)	1.94 (1.48–2.54)	**<0.001**
Injection drug use												
No	653	246 (37.7)	1		413	135 (32.7)	1		240	111 (46.3)	1	
Yes	202	95 (47.0)	1.47 (1.13–1.91)	**0.004**	127	59 (46.5)	1.79 (1.30–2.46)	**<0.001**	75	36 (48.0)	1.07 (0.86–1.33)	0.526
Prior treatment for alcohol use disorder												
No	753	280 (37.2)	1		477	157 (32.9)	1		276	123 (44.6)	1	
Yes	102	61 (59.8)	2.51 (1.64–3.84)	**<0.001**	63	37 (58.7)	2.90 (1.99–4.23)	**<0.001**	39	24 (61.5)	1.99 (1.01–3.91)	**0.045**
Prior treatment for drug use disorder												
No	336	116 (34.5)	1		206	66 (32.0)	1		130	50 (38.5)	1	
Yes	519	225 (43.4)	1.45 (0.94–2.23)	0.091	334	128 (38.3)	1.32 (0.95–1.83)	0.098	185	97 (52.4)	1.76 (0.97–3.20)	0.063
Polysubstance use^c^												
No	190	69 (36.3)	1		119	39 (32.8)	1		71	30 (42.3)	1	
Yes	665	272 (40.9)	1.21 (0.72–2.05)	0.469	421	155 (36.8)	1.20 (0.59–2.43)	0.622	244	117 (48.0)	1.26 (0.80–1.98)	0.316

*Note*: Bolded values indicate statistically significant associations (*P* < 0.05).

Abbreviations: ASI-Psych, Addiction Severity Index version 5; GED, General Education Development; PD, psychological distress.

a Estimates are unadjusted/univariable.

b GED or GED certification is equivalent to a high school diploma in the United States.

c The number of various substances, drawn from the ASI drug domain, was defined as using at least two categories of substances (including the target drug—methamphetamine) in the past 30 days. The substance categories included alcohol, heroin, other opiates or analgesics, barbiturates, other sedative-hypnotics or tranquilizers, cocaine, cannabis, hallucinogens and inhalants.

**TABLE 2:** The association of psychological distress with three outcomes of methamphetamine use disorders stratified analysis by gender.

	*n* (%)	Model A^a^		Model B^b^	
		aOR (95% CI)	*P* value	aOR (95% CI)	*P* value
Any reduced frequency of use (baseline to the EOT)					
Total					
Without PD	168 (51.4)	1		1	
With PD	105 (47.7)	0.81 (0.74–0.88)	**0.001**	0.74 (0.66–0.82)	**<0.001**
Men					
Without PD	115 (50.9)	1		1	
With PD	73 (56.0)	1.18 (0.76–1.81)	0.461	1.05 (0.69–1.62)	0.811
Women					
Without PD	53 (52.6)	**1**			
With PD	32 (35.8)	0.44 (0.26–0.74)	**0.002**	0.41 (0.23–0.75)	**0.003**
Positive urine test for methamphetamine at EOT					
Total sample					
Without PD	205 (62.0)	1		1	
With PD	154 (64.5)	1.16 (1.04–1.30)	**0.008**	1.27 (1.08–1.49)	**0.004**
Men					
Without PD	137 (60.0)	1		1	
With PD	77 (55.1)	0.84 (0.57–1.22)	0.353	1.00 (0.69–1.44)	0.991
Women					
Without PD	68 (65.3)	1		1	
With PD	77 (78.8)	2.41 (1.52–3.81)	**<0.001**	2.18 (1.25–3.81)	**0.006**
Positive urine test for other drug use^c^ at EOT					
Total sample					
Without PD	122 (38.9)	1		1	
With PD	101 (43.2)	1.16 (0.75–1.78)	0.511	1.17 (0.85–1.63)	0.338
Men					
Without PD	85 (39.5)	1		1	
With PD	45 (32.2)	0.63 (0.41–0.97)	**0.034**	0.60 (0.45–0.80)	**<0.001**
Women					
Without PD	37 (36.2)	1		1	
With PD	56 (60.6)	2.93 (1.43–5.99)	**0.003**	3.16 (1.53–6.47)	**<0.001**

*Note*: Bolded values indicate statistically significant associations (*P* < 0.05).

Abbreviations: aOR, adjusted odds ratio; EOT, end of trial; ID, identification; IPW, inverse probability weighting; PD, psychological distress.

a In model A, the logistic regression models were adjusted for demographic and clinical covariates and included clustering study ID to consider heterogeneity between studies.

b In model B, in addition to adjustment for the covariates and clustering study ID, the missingness in outcome measures was addressed using IPW.

c Other drugs include cocaine, opioids, benzodiazepines, cannabis and barbiturates.

**TABLE 3:** Interaction of psychological distress at baseline with treatment effect on three outcomes of MUD.

	Any reduced use			Positive urine test for methamphetamine			Positive urine test for other drugs		
	Study arms		PD × MUD treatment interaction test^a^	Study arms		PD × MUD treatment interaction test^a^	Study arms		PD × MUD treatment interaction test^a^
	Active	Placebo		Active	Placebo		Active	Placebo	
Total									
Without PD	56.1	47.6	χ^2^ = 1.75;	57.0	66.1	χ^2^ = 2.24;	34.1	44.8	χ^2^ = 1.27;
With PD	43.4	48.1	*P* = 0.186	67.3	63.9	*P* = 0.134	41.5	42.5	*P* = 0.260
Men									
Without PD	54.6	49.6	χ^2^ = 0.29;	54.5	62.4	χ^2^ = 3.33;	35.8	44.3	χ^2^ = 1.69;
With PD	52.3	55.8	*P* = 0.593	60.0	54.7	*P* = 0.068	29.9	27.7	*P* = 0.193
Women									
Without PD	59.7	44.0	χ^2^ = 7.85;	58.7	73.1	χ^2^ = 0.20;	30.0	45.9	χ^2^ = 0.91;
With PD	31.4	35.5	***P* = 0.005**	77.1	81.4	*P* = 0.656	58.4	63.8	*P* = 0.341
Gender × PD × MUD treatment, interaction test^b^	χ^2^ = 27.89; ***P* < 0.001**			χ^2^ = 21.00; ***P* < 0.001**			χ^2^ = 143.26; ***P* < 0.001**		

*Note*: Bolded values indicate statistically significant associations (*P* < 0.05).

Abbreviations: PD, psychological distress; MUD, methamphetamine use disorder.

a The two-way interaction term includes PD with a validated cut-off ≥24.6 (yes vs. no), treatment arms. Analyses were stratified by gender.

b The three-way interaction term includes PD with a validated cut-off ≥24.6 (yes vs. no), treatment arms (active treatment vs. placebo) and gender (women vs. men).

## Data Availability

Raw data and associated documents are publicly available on the National Institute on Drug Abuse (NIDA) Data Share website (https://datashare.nida.nih.gov).

## References

[1] Substance Abuse and Mental Health Services Administration. Key substance use and mental health indicators in the United States: Results from the 2023 National Survey on Drug Use and Health, (HHS Publication No. PEP24-07-021, NSDUH Series H-59): Center for Behavioral Health Statistics and Quality, Substance Abuse and Mental Health Services Administration. https://www.samhsa.gov/data/report/2023-nsduh-annual-national-report; 2024.

[2] Scott JC, Woods SP, Matt GE, Meyer RA, Heaton RK, Atkinson JH, Neurocognitive effects of methamphetamine: A critical review and meta-analysis. Neuropsychol Rev. 2007;17(3):275–97. 10.1007/s11065-007-9031-0 17694436

[3] Tobolski J, Sawyer DB, Song SJ, Afari ME. Cardiovascular disease associated with methamphetamine use: a review. Heart Fail Rev. 2022;27(6):2059–65. 10.1007/s10741-022-10261-7 35844009

[4] Salamanca SA, Sorrentino EE, Nosanchuk JD, Martinez LR. Impact of methamphetamine on infection and immunity. Front Neurosci. 2015;8:445. 25628526 10.3389/fnins.2014.00445 PMC4290678

[5] Corsi KF, Booth RE. HIV sex risk behaviors among heterosexual methamphetamine users: literature review from 2000 to present. Curr Drug Abuse Rev. 2008;1(3):292–6. 10.2174/1874473710801030292 19630727

[6] Halkitis PN, Parsons JT, Stirratt MJ. A double epidemic: Crystal methamphetamine drug use in relation to HIV transmission among gay men. J Homosex. 2001;41(2):17–35. 10.1300/J082v41n02_02 11482426

[7] Glasner-Edwards S, Mooney LJ, Marinelli-Casey P, Hillhouse M, Ang A, Rawson RA. Psychopathology in methamphetamine-dependent adults 3 years after treatment. Drug Alcohol Rev. 2010;29(1):12–20. 10.1111/j.1465-3362.2009.00081.x 20078677 PMC3772133

[8] Glasner-Edwards S, Mooney LJ, Marinelli-Casey P, Hillhouse M, Ang A, Rawson R. Anxiety disorders among methamphetamine dependent adults: Association with post-treatment functioning. Am J Addict. 2010;19(5):385–90. 10.1111/j.1521-0391.2010.00061.x 20716300 PMC3159418

[9] Glasner-Edwards S, Mooney LJ, Marinelli-Casey P, Hillhouse M, Ang A, Rawson R. Clinical course and outcomes of methamphetamine-dependent adults with psychosis. J Subst Abuse Treat. 2008;35(4):445–50. 10.1016/j.jsat.2007.12.004 18294802

[10] Mcketin R, Lubman DI, Lee NM, Ross JE, Slade TN. Major depression among methamphetamine users entering drug treatment programs. Med J Aust. 2011;195(3):S51–5. 10.5694/j.1326-5377.2011.tb03266.x 21806520

[11] Greenfield SF, Back SE, Lawson K, Brady KT. Substance Abuse in Women. Psychiatr Clin N Am. 2010;33(2):339–55. 10.1016/j.psc.2010.01.004 PMC3124962 20385341

[12] Booth BM, Leukefeld C, Falck R, Wang J, Carlson R. Correlates of rural methamphetamine and cocaine users: results from a multistate community study. J Stud Alcohol. 2006;67(4):493–501. 10.15288/jsa.2006.67.493 16736068

[13] Watson RJ, Caba AE, Layland EK, Simon K, Morgan E, Edelman EJ, Co-occurring mental health and drug use experiences among Black and Hispanic/Latino sexual and gender diverse individuals. J Behav Med. 2023;46(6):986–95. 10.1007/s10865-023-00433-7 37407904 PMC12004359

[14] Scott LA, Roxburgh A, Bruno R, Matthews A, Burns L. The impact of comorbid cannabis and methamphetamine use on mental health among regular ecstasy users. Addict Behav. 2012;37(9):1058–62. 10.1016/j.addbeh.2012.04.012 22607716

[15] Crouse JJ, Lee RSC, White D, Moustafa AA, Hickie IB, Hermens DF. Distress and sleep quality in young amphetamine-type stimulant users with an affective or psychotic illness. Psychiatry Res. 2018; 262:254–61. 10.1016/j.psychres.2018.02.033 29475104

[16] Parisi A, Landicho HL, Hudak J, Leknes S, Froeliger B, Garland EL. Emotional distress and pain catastrophizing predict cue-elicited opioid craving among chronic pain patients on long-term opioid therapy. Drug Alcohol Depend. 2022;233:109361. 10.1016/j.drugalcdep.2022.109361 35278786 PMC9466292

[17] Brady KT, Sinha R. Co-Occurring Mental and Substance Use Disorders: The Neurobiological Effects of Chronic Stress. Am J Psychiatry. 2005;162(8):1483–93. 10.1176/appi.ajp.162.8.1483 16055769

[18] Sinha R, Fuse T, Aubin LR, O’malley SS. Psychological stress, drug-related cues and cocaine craving. Psychopharmacology (Berl). 2000;152(2):140–8. 10.1007/s002130000499 11057517

[19] Rounsaville BJ. Treatment of cocaine dependence and depression. Biol Psychiatry. 2004;56(10):803–9. 10.1016/j.biopsych.2004.05.009 15556126

[20] Glasner-Edwards S, Marinelli-Casey P, Hillhouse M, Ang A, Mooney LJ, Rawson R. Depression among methamphetamine users: Association with outcomes from the Methamphetamine Treatment Project at 3-year follow-up. J Nerv Ment Dis. 2009;197(4):225–31. 10.1097/NMD.0b013e31819db6fe 19363377 PMC2749575

[21] Polcin DL, Korcha R, Bond J, Galloway G, Nayak M. Changes in psychiatric symptoms among persons with methamphetamine dependence predicts changes in severity of drug problems but not frequency of use. Subst Abus. 2016;37(1):209–14. 10.1080/08897077.2015.1015701 25775225 PMC4573376

[22] Bornovalova MA, Gratz KL, Daughters SB, Hunt ED, Lejuez CW. Initial RCT of a distress tolerance treatment for individuals with substance use disorders. Drug Alcohol Depend. 2012;122(1):70–6. 10.1016/j.drugalcdep.2011.09.012 21983476 PMC3288895

[23] Angarita GA, Hadizadeh H, Cerdena I, Potenza MN. Can pharmacotherapy improve treatment outcomes in people with co-occurring major depressive and cocaine use disorders? Expert Opin Pharmacother. 2021;22(13):1669–83. 10.1080/14656566.2021.1931684 34042556 PMC8440354

[24] Dluzen DE, Liu B. Gender differences in methamphetamine use and responses: A review. Gend Med. 2008;5(1):24–35. 10.1016/S1550-8579(08)80005-8 18420163

[25] Balmori A, Macías A, De La Puente MP. Hormonal differences between women and men, their consequences on addiction to substances and considerations on the therapeutic approach. Curr Addict Rep. 2022;9(2):86–98. 10.1007/s40429-022-00409-8

[26] Van Der Plas EA, Crone EA, Van Den Wildenberg WPM, Tranel D, Bechara A. Executive control deficits in substance-dependent individuals: A comparison of alcohol, cocaine, and methamphetamine and of men and women. J Clin Exp Neuropsychol. 2009;31(6):706–19. 10.1080/13803390802484797 19037812 PMC2829119

[27] Lynch WJ. Sex differences in vulnerability to drug self-administration. Exp Clin Psychopharmacol. 2006;14(1):34–41. 10.1037/1064-1297.14.1.34 16503703

[28] Brady KT, Randall CL. Gender differences in substance use disorders. Psychiatr Clin N Am. 1999;22(2):241–52. 10.1016/S0193-953X(05)70074-5 10385931

[29] Zhang Y, Lu C, Zhang J, Hu L, Song H, Li J, Gender differences in abusers of amphetamine-type stimulants and ketamine in southwestern China. Addict Behav. 2013;38(1):1424–30. 10.1016/j.addbeh.2012.06.024 23006246

[30] Hartwell EE, Moallem NR, Courtney KE, Glasner-Edwards S, Ray LA. Sex Differences in the Association Between Internalizing Symptoms and Craving in Methamphetamine Users. J Addict Med. 2016;10(6): 395–401. 10.1097/ADM.0000000000000250 27504928 PMC5083163

[31] Choi S, Adams SM, Morse SA, Macmaster S. Gender differences in treatment retention among individuals with co-occurring substance abuse and mental health disorders. Subst Use Misuse. 2015;50(5):653–63. 10.3109/10826084.2014.997828 25587672

[32] Kelly SM, Schwartz RP, O’grady KE, Mitchell SG, Reisinger HS, Peterson JA, Gender differences among in-and out-of-treatment opioid-addicted individuals. Am J Drug Alcohol Abuse. 2009;35(1):38–42. 10.1080/00952990802342915 19152205 PMC2938871

[33] Greenfield SF, Brooks AJ, Gordon SM, Green CA, Kropp F, Mchugh RK, Substance abuse treatment entry, retention, and outcome in women: A review of the literature. Drug Alcohol Depend. 2007;86(1):1–21. 10.1016/j.drugalcdep.2006.05.012 16759822 PMC3532875

[34] Tuchman E Women and addiction: The importance of gender issues in substance abuse research. J Addict Dis. 2010;29(2):127–38. 10.1080/10550881003684582 20407972

[35] Elkashef A, Kahn R, Yu E, Iturriaga E, Li SH, Anderson A, Topiramate for the treatment of methamphetamine addiction: A multicenter placebo-controlled trial. Addiction. 2012;107(7):1297–306. 10.1111/j.1360-0443.2011.03771.x 22221594 PMC3331916

[36] Anderson AL, Li SH, Biswas K, Mcsherry F, Holmes T, Iturriaga E, Modafinil for the treatment of methamphetamine dependence. Drug Alcohol Depend. 2012;120(1–3):135–41. 10.1016/j.drugalcdep.2011.07.007 21840138 PMC3227772

[37] Anderson AL, Li SH, Markova D, Holmes TH, Chiang N, Kahn R, Bupropion for the treatment of methamphetamine dependence in non-daily users: A randomized, double-blind, placebo-controlled trial. Drug Alcohol Depend. 2015;150:170–4. 10.1016/j.drugalcdep.2015.01.036 25818061 PMC4388163

[38] Johnson BA, Ait-Daoud N, Elkashef AM, Smith EV, Kahn R, Vocci F, A preliminary randomized, double-blind, placebo-controlled study of the safety and efficacy of ondansetron in the treatment of methamphetamine dependence. Int J Neuropsychopharmacol. 2008;11(1):1–14. 10.1017/S1461145707007778 17470315

[39] Susukida R, Amin-Esmaeili M, Mayo-Wilson E, Mojtabai R. Data management in substance use disorder treatment research: Implications from data harmonization of National Institute on Drug Abuse funded randomized controlled trials. Clin Trials. 2020;18(2):215–25. 10.1177/1740774520972687 33258697

[40] Elkashef AM, Rawson RA, Anderson AL, Li S-H, Holmes T, Smith EV, Bupropion for the treatment of methamphetamine dependence. Neuropsychopharmacology. 2008;33(5):1162–70. 10.1038/sj.npp.1301481 17581531

[41] Mcgahan PL, Griffith JA, Parente R, Mclellann AT. Composite scores manual Treatment Research Institute; 1986.

[42] Susukida R, Mojtabai R, Amin-Esmaeili M. Validation of Addiction Severity Index (ASI) for assessment of psychiatric comorbidity in multi-site randomized controlled trials. J Dual Diagn. 2020;16(3):312–21. 10.1080/15504263.2020.1741755 32254003

[43] Leonhard C, Mulvey K, Gastfriend DR, Shwartz M. The Addiction Severity Index: a field study of internal consistency and validity. J Subst Abuse Treat. 2000;18(2):129–35. 10.1016/S0740-5472(99)00025-2 10716096

[44] Mäkelä K Studies of the reliability and validity of the Addiction Severity Index. Addiction. 2004;99(4):398–410. 15049734 10.1111/j.1360-0443.2003.00665.x

[45] Hser YI, Evans E, Huang YC. Treatment outcomes among women and men methamphetamine abusers in California. J Subst Abuse Treat. 2005;28(1):77–85. 10.1016/j.jsat.2004.10.009 15723735

[46] Roos CR, Nich C, Mun CJ, Babuscio TA, Mendonca J, Miguel AQC, Clinical validation of reduction in cocaine frequency level as an endpoint in clinical trials for cocaine use disorder. Drug Alcohol Depend. 2019;205:107648. 10.1016/j.drugalcdep.2019.107648 31677490 PMC6910212

[47] Cacciola JS, Alterman AI, Mclellan AT, Lin YT, Lynch KG. Initial evidence for the reliability and validity of a “Lite” version of the Addiction Severity Index. Drug Alcohol Depend. 2007;87(2–3):297–302. 10.1016/j.drugalcdep.2006.09.002 17045423

[48] Mclellan AT, Luborsky L, Cacciola J, Griffith J, Evans F, Barr HL, New data from the Addiction Severity Index. Reliability and validity in three centers. J Nerv Ment Dis. 1985;173(7):412–23. 10.1097/00005053-198507000-00005 4009158

[49] Zanis DA, Mclellan AT, Cnaan RA, Randall M. Reliability and validity of the Addiction Severity Index with a homeless sample. J Subst Abuse Treat. 1994;11(6):541–8. 10.1016/0740-5472(94)90005-1 7884837

[50] Amin-Esmaeili M, Farokhnia M, Susukida R, Leggio L, Johnson RM, Crum RM, Reduced drug use as an alternative valid outcome in individuals with stimulant use disorders: Findings from 13 multisite randomized clinical trials. Addiction. 2024;119(5):833–43. 10.1111/add.16409 38197836 PMC11009085

[51] Amin-Esmaeili M, Susukida R, Johnson RM, Farokhnia M, Crum RM, Thrul J, Patterns of reduced use and abstinence in multi-site randomized controlled trials of pharmacotherapies for cocaine and methamphetamine use disorders. Drug Alcohol Depend. 2021;226: 108904. 10.1016/j.drugalcdep.2021.108904 34304121 PMC12258494

[52] Burke DL, Ensor J, Riley RD. Meta-analysis using individual participant data: one-stage and two-stage approaches, and why they may differ. Stat Med. 2017;36(5):855–75. 10.1002/sim.7141 27747915 PMC5297998

[53] Debray TP, Moons KG, Van Valkenhoef G, Efthimiou O, Hummel N, Groenwold RH, Get real in individual participant data (IPD) meta-analysis: A review of the methodology. Res Synth Methods. 2015;6(4):293–309. 10.1002/jrsm.1160 26287812 PMC5042043

[54] Abo-Zaid G, Guo B, Deeks JJ, Debray TP, Steyerberg EW, Moons KG, Individual participant data meta-analyses should not ignore clustering. J Clin Epidemiol. 2013;66(8):865–73. 23651765 10.1016/j.jclinepi.2012.12.017 PMC3717206

[55] Booth BM, Curran G, Han X, Wright P, Frith S, Leukefeld C, Longitudinal relationship between psychological distress and multiple substance use: results from a three-year multisite natural-history study of rural stimulant users. J Stud Alcohol Drugs. 2010;71(2):258–67. 10.15288/jsad.2010.71.258 20230724 PMC2841737

[56] Hassel A, Nordfjærn T, Hagen R. Psychological and interpersonal distress among patients with substance use disorders: Are these factors associated with continued drug use and do they change during treatment? J Subst Use. 2013;18(5):363–76. 10.3109/14659891.2012.685122

[57] Mcketin R, Leung J, Stockings E, Huo Y, Foulds J, Lappin JM, Mental health outcomes associated with of the use of amphetamines: A systematic review and meta-analysis. EClinicalMedicine. 2019;16:81–97. 10.1016/j.eclinm.2019.09.014 31832623 PMC6890973

[58] Compton WM III, Cottler LB, Ben Abdallah A, Phelps DL, Spitznagel EL, Horton JC. Substance dependence and other psychiatric disorders among drug dependent subjects: Race and gender correlates. Am J Addict. 2000;9(2):113–25. 10934573 10.1080/10550490050173181

[59] Green KM, Zebrak KA, Robertson JA, Fothergill KE, Ensminger ME. Interrelationship of substance use and psychological distress over the life course among a cohort of urban African Americans. Drug Alcohol Depend. 2012;123(1–3):239–48. 10.1016/j.drugalcdep.2011.11.017 22189347 PMC3319235

[60] Lee HY, Hwang J, Ball JG, Lee J, Yu Y, Albright DL. Mental Health Literacy Affects Mental Health Attitude: Is There a Gender Difference? Am J Health Behav. 2020;44(3):282–91. 10.5993/AJHB.44.3.1 32295676

[61] Wendt D, Shafer K. Gender and attitudes about mental health help seeking: Results from national data. Health Soc Work. 2015;41(1):e20–8. 10.1093/hsw/hlv089

[62] Agterberg S, Schubert N, Overington L, Corace K. Treatment barriers among individuals with co-occurring substance use and mental health problems: Examining gender differences. J Subst Abuse Treat. 2020;112:29–35. 32199543 10.1016/j.jsat.2020.01.005

[63] Susukida R, Mojtabai R, Mendelson T. Sex Differences in Help Seeking for Mood and Anxiety Disorders in the National Comorbidity Survey-Replication. Depress Anxiety. 2015;32(11):853–60. 10.1002/da.22366 25903117

[64] Compton WM III, Cottler LB, Jacobs JL, Ben-Abdallah A, Spitznagel EL. The role of psychiatric disorders in predicting drug dependence treatment outcomes. Am J Psychiatry. 2003;160(5):890–5. 12727692 10.1176/appi.ajp.160.5.890

[65] Daigre C, Rodríguez L, Roncero C, Palma-Álvarez RF, Perea-Ortueta M, Sorribes-Puertas M, Treatment retention and abstinence of patients with substance use disorders according to addiction severity and psychiatry comorbidity: A six-month follow-up study in an outpatient unit. Addict Behav. 2021;117:106832. 33529849 10.1016/j.addbeh.2021.106832

[66] Compton Iii WM, Cottler LB, Jacobs JL, Ben-Abdallah A, Spitznagel EL. The Role of Psychiatric Disorders in Predicting Drug Dependence Treatment Outcomes. Am J Psychiatry. 2003;160(5):890–5. 10.1176/appi.ajp.160.5.890 12727692

[67] Najt P, Fusar-Poli P, Brambilla P. Co-occurring mental and substance abuse disorders: a review on the potential predictors and clinical outcomes. Psychiatry Res. 2011;186(2–3):159–64. 10.1016/j.psychres.2010.07.042 20728943

[68] Byregowda H, Amin-Esmaeili M, Susukida R, Mojtabai R, Crum R. Association between psychiatric comorbidity and retention in substance use disorder treatment: Results from multi-site randomized controlled trials of pharmacological and behavioral interventions. J Dual Diagn. 2025 In press). 267:111761. 10.1016/j.drugalcdep.2024.111761 PMC12961725 41478298

[69] Korte JE, Rosa CL, Wakim PG, Perl HI. Addiction treatment trials: How gender, race/ethnicity, and age relate to ongoing participation and retention in clinical trials. Subst Abuse Rehabil. 2011;2:205–18. 24474858 10.2147/SAR.S23796 PMC3846502

