# Supplementary material for: Gender differences in the impact of psychological distress on methamphetamine use disorder outcomes and treatment effect
Source: Addiction. Author manuscript; Available in PMC 2026 Mar 6. (PMC12961701; doi:10.1111/add.70315)
Supplement: Appendix S1. Supplementary Information on statitiscal analysis [file NIHMS2143956-supplement-Appendix_S1__Supplementary_Information_on_statitiscal_analysis.docx]

# **Detailed description of statistical analysis**

Analyses were conducted in four steps.
Step 1: Baseline sociodemographic and clinical characteristics were summarized for participants with and without psychological distress (PD) and compared using univariable logistic regression analyses. Because subsequent regression models detected significant PD × gender interactions, baseline characteristics were also summarized separately for women and men.

Step 2: We compared PD symptoms from ASI-Psych items between women and men using Fisher’s exact tests for low-frequency items (e.g., suicide attempts) and chi-square tests for all other items. Treatment retention (completion of the active treatment phase) was similarly compared among groups identified by PD status and gender.

Step 3: Following established one-stage IPD meta-analysis guidelines [52-54], we estimated the association of PD with three outcomes: (1) reduction in methamphetamine use frequency, (2) methamphetamine-positive urine toxicology at trial end, and (3) urine toxicology–verified use of other drugs. PD was modeled as a binary variable (ASI-Psych ≥ 24.6) in primary analyses to enhance clinical interpretability. Sensitivity analyses treated PD as a continuous ASI-Psych composite score (0–100). Analyses were first conducted in the full sample. Due to evidence of effect modification by gender, all models were repeated in gender-stratified samples. PD was conceptualized as both a prognostic predictor and a moderator of treatment effects. Because PD was measured at baseline, it could not be evaluated as a mediator. Interaction terms for PD × treatment were included to assess whether active pharmacological treatment effects differed by PD status.

Two regression models were estimated for each outcome. Model A adjusted for sociodemographic and clinical covariates, including gender (women, men), age, race/ethnicity (non-Hispanic White vs. other), marital status (legally married/cohabiting, divorced/widowed/separated, single/never married), education (high school diploma/General Educational Development [GED] certificate—which is equivalent to a high school diploma in the US—vs. higher education), history of chronic medical illness (yes, no), injecting drug use (yes, no), prior treatment for alcohol or drug use disorders (yes, no), and polysubstance use (use of at least two substance categories, including the target drug—methamphetamine) in the past 30 days (yes, no), and length of trial. To account for within-trial correlations, we used cluster-robust standard errors at the trial level, which provide population-average inference while allowing for correlated observations within each trial. Model B further addressed missing outcome data using inverse probability weighting (IPW), in which the probability of missingness was modeled using observed covariates, and complete cases were weighted accordingly. Logistic regression models generated odds ratios (ORs) with 95% confidence intervals (CIs). All models were repeated in gender-stratified analyses.

Step 4: To evaluate potential moderation of treatment efficacy by gender and PD, we tested three-way interaction terms (treatment × gender × PD) using Stata’s factor-variable notation (e.g., i.treatment##i.gender##i.PD). Models also included relevant two-way interactions (treatment × gender; treatment × PD; gender × PD) and main effects for each variable. Wald tests were used to assess the significance of the three-way interaction. Each interaction term was examined separately for each outcome. As additional exploration, we also conducted analyses stratified by gender. All models were adjusted for sociodemographic and clinical covariates, study ID, and missing outcome data. Analyses were performed in Stata 18.5 using a significance level of p < 0.05.
