# Supplementary material for: Gender differences in the impact of psychological distress on methamphetamine use disorder outcomes and treatment effect
Source: Addiction. Author manuscript; Available in PMC 2026 Mar 6. (PMC12961701; doi:10.1111/add.70315)
Supplement: Figure S1. Flowchart of selected RCTs of pharmacotherapies for methamphetamine use disorders (MUD) [file NIHMS2143956-supplement-Figure_S1__Flowchart_of_selected_RCTs_of_pharmacotherapies_for_methamphetamine_use_disorders__MUD_.docx]

Total studies on the NIDA Data Share website (N=83)

RCTs of pharmacotherapeutic interventions on methamphetamine use disorders (n=13)

Phase 1 RCTs (feasibility/interaction studies) (n=6)

Lack of core clinical measures (n=2)

Included RCTs on methamphetamine use disorder (n=5)

**FIGURE S1.** Flowchart of selected RCTs of pharmacotherapies for methamphetamine use disorders (MUD)
