# Supplementary material for: Gender differences in the impact of psychological distress on methamphetamine use disorder outcomes and treatment effect
Source: Addiction. Author manuscript; Available in PMC 2026 Mar 6. (PMC12961701; doi:10.1111/add.70315)
Supplement: Supplementary Tables S1 and S2 [file NIHMS2143956-supplement-Supplementary_Tables_S1_and_S2.docx]

**TABLE S1** Sociodemographic, clinical, and drug use correlates of psychological distress (score of ASI-Psych; 0-100) at baseline, by gender

|  | **ASI-Psych composite score (0-100)** | | | | | | | | | | | |
| --- | --- | --- | --- | --- | --- | --- | --- | --- | --- | --- | --- | --- |
|  | **Total sample** | | | | | **Men** | | |  | **Women** | | |
|  | Mean (SE) | Coeff (95% CI)^a^ | P value |  | Mean (SE) | | Coeff (95% CI) ^a^ | P value |  | Mean (SE) | Coeff (95% CI) ^a^ | P value |
| **Age, years** |  |  |  |  |  | |  |  |  |  |  |  |
| <35 | 24.4 (1.3) | 1 |  |  | 22.7 (1.5) | | 1 |  |  | 27.3 (2.1) | 1 |  |
| 35-45 | 19.7 (1.1) | -4.74 (-8.38, -1.10) | **0.022** |  | 17.3 (1.3) | | -5.36 (-9.48, -1.24) | **0.022** |  | 23.5 (1.9) | -3.80 (-8.53, 0.94) | 0.090 |
| >45 | 17.3 (1.5) | -7.11 (-12.78, -1.45) | **0.025** |  | 15.1 (1.7) | | -7.5 (-13.24, -1.79) | **0.022** |  | 21.8 (2.9) | -5.56 (-14.55, 3.43) | 0.161 |
| **Race/ethnicity** |  |  |  |  |  | |  |  |  |  |  |  |
| Other/multiple | 18.6 (1.3) | 1 |  |  | 18.4 (1.5) | | 1 |  |  | 19.2 (2.5) | 1 |  |
| Non-Hispanic White | 21.9 (0.8) | 3.26 (0.73, 6.36) | **0.023** |  | 18.9 (1.1) | | 0.56 (-2.08, 3.19) | 0.588 |  | 26.3 (1.4) | 7.07 (0.79, 13.34) | **0.035** |
| **Unemployed** |  |  |  |  |  | |  |  |  |  |  |  |
| No | 20.8 (0.8) | 1 |  |  | 18.8 (0.9) | | 1 |  |  | 24.9 (1.5) | 1 |  |
| Yes | 21.7 (1.7) | 0.87 (-4.13, 5.87) | 0.654 |  | 18.7 (2.4) | | -0.08 (-7.86, 7.69) | 0.978 |  | 24.1 (2.4) | -0.78 (-4.41, 2.86) | 0.586 |
| **Marital status** |  |  |  |  |  | |  |  |  |  |  |  |
| Legally married/ cohabitating | 20.6 (1.5) | 1 |  |  | 19.0 (1.9) | | 1 |  |  | 23.7 (2.7) | 1 |  |
| Divorced/Widowed/Separated | 22.2 (1.2) | 1.57 (-4.54, 7.68) | 0.249 |  | 20.7 (1.6) | | 1.76 (-5.07, 8.58) | 0.514 |  | 24.0 (2.0) | 0.34 (-8.07, 8.74) | 0.917 |
| Single/Never married | 20.2 (1.1) | -0.43 (-3.47, 2.62) | 0.515 |  | 17.3 (1.3) | | -1.67 (-3.84, 0.51) | 0.101 |  | 25.9 (2.1) | 2.29 (-9.53, 14.11) | 0.619 |
| **Education** |  |  |  |  |  | |  |  |  |  |  |  |
| Higher education | 18.8 (1.0) | 1 |  |  | 15.8 (1.2) | | 1 |  |  | 23.7 (1.7) | 1 |  |
| High school/GED ^b^ or lower | 22.6 (1.0) | 3.82 (2.02, 5.62) | **0.004** |  | 21.0 (1.2) | | 5.21 (0.88, 9.53) | **0.029** |  | 25.5 (1.8) | 1.80 (-7.83, 11.43) | 0.632 |
| **Chronic medical illness** |  |  |  |  |  | |  |  |  |  |  |  |
| No | 19.6 (0.8) | 1 |  |  | 17.9 (0.9) | | 1 |  |  | 22.6 (1.4) | 1 |  |
| Yes | 26.5 (1.8) | 6.89 (1.95, 11.82) | **0.018** |  | 22.5 (2.3) | | 4.55 (-0.14, 9.25) | 0.055 |  | 32.6 (2.8) | 10.01 (3.58, 16.45) | **0.012** |
| **Injection drug use** |  |  |  |  |  | |  |  |  |  |  |  |
| No | 19.7 (0.8) | 1 |  |  | 17.3 (1.0) | | 1 |  |  | 24.0 (1.4) | 1 |  |
| Yes | 24.8 (1.5) | 5.10 (0.49, 9.73) | **0.037** |  | 23.7 (1.9) | | 6.41 (3.03, 9.80) | **0.006** |  | 26.8 (2.6) | 2.83 (-4.76, 10.42) | 0.359 |
| **Prior treatment for alcohol use disorder** | | | | | | | | | | | | |
| No | 20.0 (0.8) | 1 |  |  | 17.7 (0.9) | | 1 |  |  | 23.9 (1.3) | 1 |  |
| Yes | 28.2 (2.1) | 8.23 (1.37, 15.09) | **0.029** |  | 26.8 (2.7) | | 9.14 (1.11, 17.18) | **0.034** |  | 30.4 (3.5) | 6.50 (-2.71, 15.71) | 0.122 |
| **Prior treatment for drug use disorder** | | | | | | | | | | | | |
| No | 18.2 (1.1) | 1 |  |  | 16.4 (1.4) | | 1 |  |  | 21.2 (1.8) | 1 |  |
| Yes | 22.7 (1.0) | 4.44 (-2.00, 10.88) | 0.128 |  | 20.2 (1.1) | | 3.84 (-1.54, 9.21) | 0.119 |  | 27.1 (1.7) | 5.94 (-2.41, 14.29) | 0.120 |
| **Polysubstance use ^c^** |  |  |  |  |  | |  |  |  |  |  |  |
| No | 17.4 (1.4) | 1 |  |  | 14.9 (1.8) | | 1 |  |  | 21.7 (2.3) | 1 |  |
| Yes | 21.9 (0.8) | 4.49 (-2.59, 11.60) | 0.153 |  | 19.9 (1.0) | | 4.95 (-4.09, 14.00) | 0.203 |  | 25.5 (1.5) | 3.83 (-1.78, 9.43) | 0.131 |

**Notes:** CI=Confidence Interval; OR= Odds Ratio.

^a^ Estimates are unadjusted/univariable.

^b^ GED: General Education Development or GED certification is equivalent to a high school diploma in the US.

^c^ Number of various substances, drawn from ASI drug domain, were defined as using at least two categories of substances (including target drug—methamphetamine) in the past 30 days. The categories included alcohol, heroin, other opiates or analgesics, barbiturates, other sedative-hypnotics or tranquilizers, cocaine, cannabis, hallucinogens, and inhalants.

**TABLE S2** The association of psychological distress (score of ASI-Psych; 0-100) and three outcomes of methamphetamine use disorders, stratified analysis by gender

|  | **ASI-Psych composite score (0-100)** | | | | |
| --- | --- | --- | --- | --- | --- |
|  | Model A^a^ | |  | Model B^b^ | |
|  | Odds ratio (95% CI) | p value |  | Odds ratio (95% CI) | p value |
| **Any reduced frequency of use (baseline to the EOT)** |  |  |  |  |  |
| Total sample | 0.996 (0.992, 1.001) | 0.145 |  | 0.995 (0.990, 0.999) | **0.033** |
| Men | 1.001 (0.990, 1.011) | 0.919 |  | 0.998 (0.989, 1.009) | 0.759 |
| Women | 0.990 (0.977, 1.003) | 0.141 |  | 0.990 (0.976, 1.003) | 0.141 |
| **Positive urine test for methamphetamine at EOT** |  |  |  |  |  |
| Total sample | 1.003 (1.000, 1.007) | 0.056 |  | 1.006 (1.003, 1.008) | **<0.001** |
| Men | 0.997 (0.991, 1.003) | 0.329 |  | 1.001 (0.994, 1.007) | 0.767 |
| Women | 1.017 (1.004, 1.029) | **0.009** |  | 1.015 (1.002, 1.028) | **0.023** |
| **Positive urine test for other drug use at EOT ^c^** |  |  |  |  |  |
| Total sample | 1.005 (0.998, 1.012) | 0.171 |  | 1.004 (0.998, 1.011) | 0.216 |
| Men | 0.993 (0.986, 1.001) | 0.092 |  | 0.991 (0.983, 0.999) | **0.029** |
| Women | 1.022 (1.007, 1.037) | **0.003** |  | 1.023 (1.007, 1.039) | **0.005** |

**Notes:** aOR=Adjusted odds ratio; CI=Confidence Interval; EOT= End of Trial.

^a^ In Model A, the logistic regression models were adjusted for demographic and clinical covariates and included clustering study ID to consider heterogeneity between studies.

^b^ In Model B, in addition to adjustment for demographic covariates, and clustering study ID, the missingness in outcome measures were addressed using Inverse Probability Weighting (IPW).

^c^ Other drugs include cocaine, opioids, benzodiazepines, cannabis, and barbiturates.
